# Adélie Penguin Population Diet Monitoring by Analysis of Food DNA in Scats

**DOI:** 10.1371/journal.pone.0082227

**Published:** 2013-12-16

**Authors:** Simon N. Jarman, Julie C. McInnes, Cassandra Faux, Andrea M. Polanowski, James Marthick, Bruce E. Deagle, Colin Southwell, Louise Emmerson

**Affiliations:** 1 Australian Antarctic Division, Kingston, Tasmania, Australia; 2 Menzies Research Institute Tasmania, Hobart, Tasmania, Australia; Institute of Ecology, Germany

## Abstract

The Adélie penguin is the most important animal currently used for ecosystem monitoring in the Southern Ocean. The diet of this species is generally studied by visual analysis of stomach contents; or ratios of isotopes of carbon and nitrogen incorporated into the penguin from its food. There are significant limitations to the information that can be gained from these methods. We evaluated population diet assessment by analysis of food DNA in scats as an alternative method for ecosystem monitoring with Adélie penguins as an indicator species. Scats were collected at four locations, three phases of the breeding cycle, and in four different years. A novel molecular diet assay and bioinformatics pipeline based on nuclear small subunit ribosomal RNA gene (SSU rDNA) sequencing was used to identify prey DNA in 389 scats. Analysis of the twelve population sample sets identified spatial and temporal dietary change in Adélie penguin population diet. Prey diversity was found to be greater than previously thought. Krill, fish, copepods and amphipods were the most important food groups, in general agreement with other Adélie penguin dietary studies based on hard part or stable isotope analysis. However, our DNA analysis estimated that a substantial portion of the diet was gelatinous groups such as jellyfish and comb jellies. A range of other prey not previously identified in the diet of this species were also discovered. The diverse prey identified by this DNA-based scat analysis confirms that the generalist feeding of Adélie penguins makes them a useful indicator species for prey community composition in the coastal zone of the Southern Ocean. Scat collection is a simple and non-invasive field sampling method that allows DNA-based estimation of prey community differences at many temporal and spatial scales and provides significant advantages over alternative diet analysis approaches.

## Introduction

Seabirds are widely used as biological indicator species because they are abundant top-level consumers in marine food webs and their population status can be used to infer overall ecosystem status [1]–[3]. The Adélie penguin (*Pygoscelis adeliae*) is the most widely distributed Antarctic penguin [2], [4]. These penguins spend the majority of their lives in the Southern Ocean, where they are known to feed on a range of mid-sized animals such as krill, small fish, amphipods and squid [2], [5]–[8]. Adélie penguins are a useful indicator species for biological productivity because of their strong association with the pack ice, their presence at regularly-occupied breeding colonies, and their generalist diet [9]. Pack ice is one of earth’s largest continuous habitats, ranging from 1.8×10^7^ km^2^ at its winter maximum to 3×10^6^ km^2^ at the summer minimum [10], [11]. Interannual differences in pack ice extent are known to cause changes in distribution and recruitment of key Adélie penguin diet items such as Antarctic krill [11], [12]. Their suitability as an indicator species led to Adélie penguins being selected by the Commission for the Conservation of Antarctic Marine Living Resources (CCAMLR) as a key species for the CCAMLR Ecosystem Monitoring Program (CEMP) [13]. CCAMLR is the international body that manages fisheries and wildlife in the Southern Ocean. CCAMLR makes its decisions by an ecosystem-based approach, so that fisheries are managed for their impact on dependent species as well as target species [14]. Most countries with an interest in Antarctic living resources are CCAMLR members. Many of these contribute to the CEMP by collecting information on key population parameters of Adélie penguins, one of which is diet [15].

Penguin diet has generally been studied by identifying hard parts in stomach contents obtained from breeding birds by stomach flushing (Wilson 1984); and analysis of ratios of stable isotopes present in feathers, blood, eggshell [6] or various tissue taken from corpses [16]. Stomach flushing studies have provided the most detailed dietary information. They indicate that Euphausiids (krill) and small fish, primarily the Antarctic silverfish *Pleuragramma antarcticum* are the primary prey species [17]. Isotopes of carbon (δ^13^C) and nitrogen (δ^15^N) are established in animal tissues over long time periods from the mixture of isotopes present in food that they consume. Stable isotope analysis is most useful for detecting major shifts in the trophic level of species consumed by animal populations that occur over substantial blocks of time [6], [18].

The first DNA based diet analysis for Adélie penguins focused on identifying the krill species that they consumed [5]. Since then there have been numerous technical advances in DNA based diet analysis that have improved the types of dietary questions that can be answered, increased the number of samples that can be processed, and decreased the costs of analysis. DNA based diet studies of Macaroni penguins (*Eudyptes chrysolophus*) have demonstrated shifts in diet composition associated with changes in foraging strategy during different phases of the breeding cycle [19]. Studies of Little penguins (*Eudyptula minor*) demonstrated that diet data based on proportions of sequences produced by high throughput sequencers was congruent with proportions estimated by quantitative PCR [20]; although DNA-sequence based proportions are often not congruent with biomass proportions [21]. Blocking primers for suppressing amplification of predator DNA combined with PCR primers that amplify ‘barcode’ regions from a broad range of food species produce amplicon mixes that are predominately food DNA [22], [23]. Parallel sequencing technologies have become accessible for most laboratories and these allow very detailed analysis of amplicon mixes to infer food consumed by an animal [24], [25].

Adélie penguin diet is known to vary with different phases of the summer breeding season [26], [27]. During the breeding season, both parents forage for food, some of which they bring back to the colony and feed to the chicks by regurgitating directly into the chick’s mouth. The foraging trips for breeding birds take them several hundred km (generally 20–60 km) from breeding colonies [28], primarily in areas associated with the pack ice [9], [29]. Mating and chick rearing take place on land at colonies in rocky areas of the Antarctic coastline that they occupy only during the Antarctic summer. Colonies of penguins follow a largely synchronous series of breeding phases after adults return to colonies and lay eggs in late November or early December. The stages of chick rearing are: ‘Incubation’ of eggs until hatching; ‘Guard’ phase where the chick is guarded from predators by each parent in turn while the partner forages; and ‘Crèche’ phase where chicks aggregate into groups and both parents may forage simultaneously. The chick rearing process takes from 40–65 days [4].

In this study we demonstrate that broad-scale parallel sequencing-based analysis of food DNA in Adélie penguin scats can be used as a replacement for stomach flushing methods and stable isotope analysis to estimate many features of population diet. We apply this method to comparisons of Adélie penguin diet at two spatial scales, two temporal scales; and we compare diet of males and females. We identify a broad range of prey groups in Adélie penguin diet that have not been previously identified. The results indicate that this approach is well suited to Southern Ocean ecosystem monitoring and the broad applicability of the methods suggest that it would be valuable for dietary studies of any other bird or mammal indicator species.

## Materials and Methods

### Sample Collection and DNA Purification

**Figure 1:**
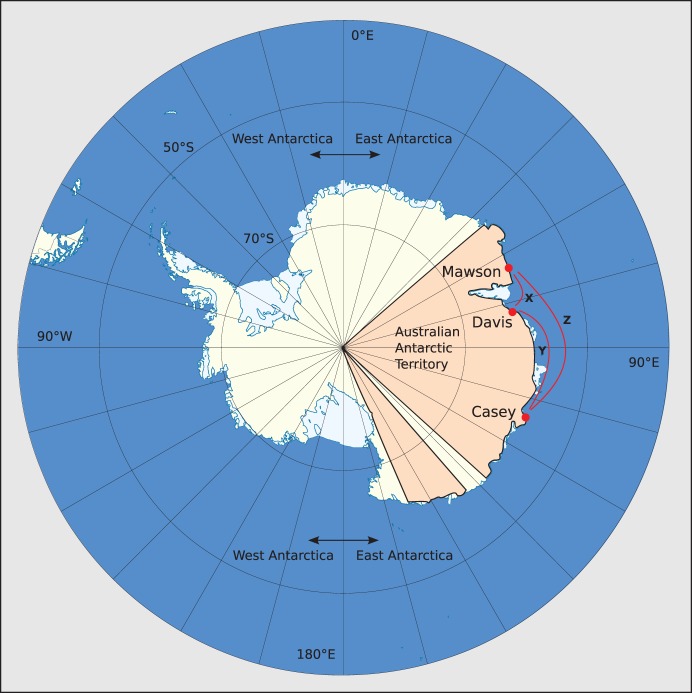
Sampling locations for Adélie penguin faeces. Sampling sites are named after the nearest Antarctic station. ‘Mawson’ samples were collected from Bechervaise Island (67°35′S, 62°49′E). ‘Davis’ samples were collected at Hop Island (68°50′S, 77°43′E). ‘Casey’ samples were collected at Whitney Point (66°16′S, 110°31′E) or at Blakeney Point (66°14′S, 110°34′E) which are 3.5 km apart. Approximate sea-route distances among sites are X ∼ 700 km, Y ∼ 1400 km and Z ∼ 2100 km.

Adélie penguin scats were collected from the areas near colonies of breeding penguins and preserved in 70–80% ethanol. Scats were collected from snow or rock between the colony and the sea. The penguins tend to follow set paths when entering or leaving the colony. It is common for them to defecate immediately after coming ashore from a foraging trip and before entering the colony. These areas were sampled frequently and all of each scat was collected, or the remainder buried to preclude repeat sampling. Sampling locations are shown in Figure 1. All scats were collected regardless of colour or form to avoid sampling bias. Samples were collected over several years and the penguin breeding season starts late in one year and finishes towards the end of the Austral summer, generally in February so samples referred to as ‘2011’ are taken from the breeding season starting in 2010 and finishing in 2011. The sample sets we analysed are shown in Table 1.

**Table 1:** Samples used in this study.

Location	Year	Breeding season phase	DNA extracted	Food amplified	Sex identified
Casey, Whitney Point	2008	Créche	64	58	48
Casey, Blakeney Point	2008	Créche	34	32	26
Casey	2012	Créche	53	33	32
Davis	2012	Incubation	19	16	16
Davis	2012	Guard	26	18	17
Davis	2012	Créche	49	35	32
Mawson	2010	Guard	50	39	34
Mawson	2010	Créche	47	39	36
Mawson	2011	Guard	50	31	26
Mawson	2011	Créche	37	32	29
Mawson	2012	Guard	53	27	25
Mawson	2012	Créche	52	29	27
**Totals = **			534	389	348

The numbers of scat samples taken at each location and time. The numbers of these samples that produced a SSU PCR product with food items in it and the number of these samples that could have their sex identified by our PCR method are also shown.

Preserved samples were stored at −20°C during transport from Antarctica. Once returned to Australia, DNA was extracted from animal scats by a Promega ‘Maxwell 16’ DNA extraction robot with a ‘Tissue DNA Purification’ kit. PCR inhibitor concentrations were reduced in the DNA extracts by a Zymogen ‘One Step™ PCR Inhibitor Removal’ kit.

### PCR Amplification of SSU rDNA

A PCR primer set for amplifying a ∼140–170 bp region of the 3′ end of the SSU rDNA of eukaryotes was designed manually on an alignment of this region that incorporated representatives of all major eukaryotic lineages. Two regions that had complete conservation in this alignment were then tested against all representatives of animal, plant and fungal phyla by BLAST searches of GenBank and minimal variation was found in the primer binding site. This primer set (SSU3′F and SSU3′R) was tested on a wide phylogenetic range of target species and was found to amplify from them as predicted. A blocking primer for suppressing amplification of penguin DNA (actually all tetrapods) was designed to bind in the region overlapping the 3′ end of SSU3′R and an adjacent region in the amplicon that is present in tetrapods, but not in other animal groups [30]. We tested this empirically as previously described [22]and found it to be successful in suppressing penguin SSU DNA amplification. This blocking primer oligo did also apparently have complete complementarity to some fish SSU rDNA, such as genbank accession JX282337for *Sarpa salpa;* and GenBank accession AF278682 for *Raja schmidti.* However, this was not the case for any of the fish groups in the Southern Ocean that are know or likely prey of Adélie penguins.

All subsequent amplifications were performed with fusion primers that incorporated the primer set described above as well as sequences for the IonTorrent sequencing system and short ‘tag’ sequences for identifying individual samples from a pooled set of sequences [31] using previously published sequence tags [32]. Each PCR contained template DNA purified from one scat, one unique combination of one of six forward and one of eight reverse primers and a ‘blocking oligo’ for suppressing amplification of penguin DNA. The primers used are shown in Table 2.

**Table 2:** Oligonucleotides used in this study.

Assay	Primer name	Primer sequence
Diet composition	SSU3′F	CACCGCCCGTCGCTACTACCG
Diet composition	SSU3′R	GGTTCACCTACGGAAACCTTGTTACG
Diet composition	TetrapodBlockC3	CCTTGTTACGACTTTTACTTCCTCTAGATAG#
Diet composition	SSU3′IonF_1	*CCATCTCATCCCTGCGTGTCTCCGACTCAG* TCAAGCCTGACACCGCCCGTCGCTACTACCG
Diet composition	SSU3′IonF_2	*CCATCTCATCCCTGCGTGTCTCCGACTCAG* CGTTAGGTCACACCGCCCGTCGCTACTACCG
Diet composition	SSU3′IonF_3	*CCATCTCATCCCTGCGTGTCTCCGACTCAG* TGCCAGGTCACACCGCCCGTCGCTACTACCG
Diet composition	SSU3′IonF_4	*CCATCTCATCCCTGCGTGTCTCCGACTCAG* CGAATGGATCCACCGCCCGTCGCTACTACCG
Diet composition	SSU3′IonF_5	*CCATCTCATCCCTGCGTGTCTCCGACTCAG* TGCCAGGACTCACCGCCCGTCGCTACTACCG
Diet composition	SSU3′IonF_6	*CCATCTCATCCCTGCGTGTCTCCGACTCAG* CAGGTAAGTCCACCGCCCGTCGCTACTACCG
Diet composition	SSU3′IonR_A	*CCTCTCTATGGGCAGTCGGTGAT* TCAGGCTTGAGGTTCACCTACGGAAACCTTGTTACG
Diet composition	SSU3′IonR_B	*CCTCTCTATGGGCAGTCGGTGAT* CGTAAGTTCAGGTTCACCTACGGAAACCTTGTTACG
Diet composition	SSU3′IonR_C	*CCTCTCTATGGGCAGTCGGTGAT* TGCAAGTTCAGGTTCACCTACGGAAACCTTGTTACG
Diet composition	SSU3′IonR_D	*CCTCTCTATGGGCAGTCGGTGAT* CGATTGAATCGGTTCACCTACGGAAACCTTGTTACG
Diet composition	SSU3′IonR_E	*CCTCTCTATGGGCAGTCGGTGAT* TGCAAGAACTGGTTCACCTACGGAAACCTTGTTACG
Diet composition	SSU3′IonR_F	*CCTCTCTATGGGCAGTCGGTGAT* CAGTTAGGTCGGTTCACCTACGGAAACCTTGTTACG
Diet composition	SSU3′IonR_G	*CCTCTCTATGGGCAGTCGGTGAT* TCGAACTTAGGGTTCACCTACGGAAACCTTGTTACG
Diet composition	SSU3′IonR_H	*CCTCTCTATGGGCAGTCGGTGAT* CTGAAGAACTGGTTCACCTACGGAAACCTTGTTACG
Sex identification	PenguinSexF	CAGCTTTAATGGAAGTGAAGG
Sex identification	PenguinSexR	GGAGTCACTATCAGATCC

‘A’ sequence, from which sequencing is primed); and the ‘P1’ sequence which binds ‘IonSpheres’ to the fusion primers. Underlined portions are tags for assigning sequences to individual scats as previously described^28^. The blocking primer used here has a 3′ C_3 spacer residue that stops it priming amplification in a PCR, which is represented by a #. Sequences of primers for two PCR assays are shown. Italics indicate the IonTorrent

PCR reactions were performed in lots of 48. Each reaction of 10 uL contained 5 uL 2 × Phusion HF (NEB), 1 × Bovine Serum Albumin (NEB), 1 uM of each amplification primer and 10 uM of blocking primer ‘TetrapodBlockC3’ (see Table 2) with the remainder of the reaction volume being scat DNA extract. PCR thermal cycling conditions were 98°C, 5 min; then 40 cycles of 98°C, 5 s; 57°C, 20 s; and 72°C, 20 s. A final extension occurred at 72°C for one minute. PCR reactions from each set were pooled and purified from unincorporated reaction components by washing utilising reversible binding to Ampure (Agencourt) magnetic beads following the manufacturer’s protocol. A negative control reaction with no template DNA was included with each batch of 48 samples and this control reaction was sequenced as well. Batches were discarded if the negative control reaction contained any sequences from diet items.

### Parallel Sequencing

Sequencing of PCR products was performed with an Ion Torrent next-generation sequencer and OneTouch semi-automated library preparation platform (Life Technology). The 200 bp sequencing kit v1 was used and 314 chips. Primary sequence estimation was done by Torrent Server software version 2.2 with the ‘Beverly read filter’ turned off to ensure the maximum number of sequences were included in the primary data. FASTQ files were transferred from the Torrent Server after checking run success based on the run reports generated by the Torrent Suite software. Samples from each location were split among different runs to avoid run-specific biases.

### Sequence Data Analysis

Sequence data was processed with scripts written in Python 2.7.2 (www.python.org) and R 2.13.1 (www.cran.r-project.org) by the authors and are available in the Dryad database archive for this paper. The Python scripts called the software package USEARCH for OTU clustering [33]. The scripts also used BLAST 2.2.6 for assigning OTUs to taxonomic categories [34]. A standalone BLAST database was created from the SILVA SSURef database release 108. This database was formatted with the full EMBL-format taxonomy used as the name for each sequence and the RNA sequences from the original database converted to DNA. It contained 5978 sequences representing most known eukaryotic lineages to genus or family level. This database is carefully curated, containing only high quality sequences derived from organisms identified by taxonomic experts [35].

Data was processed in two phases. In phase one, no aggregation of sequences to higher taxa was performed. This ‘training’ phase allowed the identification of broad prey groups that were at a taxonomic level higher than the SSU amplicon could resolve. For example, the first time a Scyphozoa sequence was identified, the closest match in our local SSU BLAST database had the full taxonomic description:

Metazoa;Cnidaria;Scyphozoa;Coronatae;Nausithoidae;Nausithoe;Nausithoe_rubra representing SSU sequence from the Scyphozoa species *Nausithoe_rubra.* At this point, we added ‘Scyphozoa’ to the list for aggregation. In any future sequence processing runs, sequences belonging to Scyphozoa were aggregated into one group in the final summaries. The software preserves each step of the processing as text files so that identifications can be tracked. All files resulting from the processing of the primary sequence data into final aggregated groups belonging to higher taxa were archived in the Dryad database entry for this paper.

After all of the samples were analysed in this training phase, a list of taxa to aggregate sequences to was generated as shown in Table 3. All of the IonTorrent runs were then re-analysed with aggregation to these higher taxa in the following steps, which follow a very similar scheme to other environmental DNA barcoding classification software such as QIIME [36]:

**Table 3:** Categories and taxa for sequence aggregation.

Food	Contaminant	Unicellular eukaryotes
Actinopterygii (Bony fish)	Aves (Birds)	Apicomplexa
Amphipoda (Amphipod shrimps)	Agaricomycotina (Fungus)	Chlamydomonadaceae
Anthomedusae (Cnidarian jelly)	Anthozoa (Sessile cnidarians)	Chromulinaceae
Asellota (Isopod shrimps)	Bacillariophyta (Diatoms)	Chrysophyceae
Bangiaceae (Red alga)	Coleoptera (Beetles)	Ciliophora
Calanoida (Calanoid copepods)	Diplopoda (Millipedes)	Ciliophora
Canalipalpata (Fan-headed worms)	Diptera (Flies)	Dinophyceae
Caryophyllales (Pearlworts)	Glomeromycetes (Fungus)	Euamoebida
Collembola (Springtails)	Neoptera (Bees)	Oomycetes
Cyclopoida (Cyclopoid copepods)	Pezizomycotina (Mold)	Rhizaria
Cephalopoda (Squid, Octopus)	Primates (Apes, monkeys, humans)	Saccharomycotina
Ctenophora (Comb jellies)	Pucciniomycotina (Fungus)	Silicofilosea
Euphausiidae (Krill)	Taphrinomycotina (Fungus)	Vannellidae
Harpacticoida (copepods)	Tardigrada (Water bears)	
Mysida (Opossum shrimps)	Turbellaria (Flatworms)	
Ostracoda (Seed shrimps)		
Palmariaceae (Red alga)	**Parasites**	
Phaeophyceae (Brown alga)	Acanthocephala (Thorny-headed worms)	
Pleocyemata (Shrimps)	Cestoda (Tapeworms)	
Pteropoda (Sea butterflies)	Monogenea (Ectoparasitic platyhelminths)	
Porifera (Sponges)	Mucoromycotina (Fungal gut parasite)	
Salpidae (Salps)	Nematoda (Roundworm)	
Scyphozoa (Jellyfish)	Oribatida (Mites)	
Siphonophora (Bluebottles)	Trematoda (Flukes)	
Siphonostomatoida (Siphonostomatoid copepods)	Trichomonadidae (Unicellular parasites)	

‘food’, ‘parasite’, ‘contaminant’, ‘unicellular eukaryotes’). The taxonomic levels to which groups of related sequences were aggregated within these categories are given as well as their common names. Four categories of sequences items were determined (

1. FASTQ files were first filtered for quality with any sequence not having a mean Q score of 30 being discarded. A minimum length cut-off of 120 bp applied to remove partial reads, primer-dimer and short, non-target amplicons, resulting in one large FASTA file per sequencing run.
2. The overall FASTA file was divided into files containing sequences derived from individual scats based on presence of unique sequence barcode combinations present in the forward and reverse primers.
3. Sequences within each scat-specific file were grouped by similarity using USEARCH with a similarity cut-off of 90%. This cut-off was chosen after empirical experimentation as one that separated sequences into the categories determined after the training phase.
4. ‘Seed’ sequences representing each 90%-similar group of sequences were used to query a local BLAST database of SSU sequences. Any sequences that did not have a BLAST match were discarded, which would remove chimaeric sequences, but probably also some sequences that are not represented in the database such as some basal eukaryotic lineages.
5. Sequences attaining a match of >90% identity to a database entry were aggregated to a higher taxon and category as determined by the list in Table 3. Sequences that did not match any pre-defined higher taxon were reported to their closest species match in the database (this reporting was used entirely in the training phase).
6. Scats that returned fewer than 50 food sequences were removed from analysis. Proportions of food items in each pre-defined higher taxon were determined and population averages calculated as a mean of proportions per scat.

The assignment of sequences to categories started with a list of known Adélie penguin prey groups such as Euphausiidae and Actinopterygii for the ‘food category’; and lists of known contaminating taxa such as Primates (because human DNA is an inevitable contaminant), Diptera (flies) and Pezizomycotina (including many mold fungi) that represent laboratory contamination rather than penguin diet items for the ‘contaminant’ category. These lists were primarily derived from the ‘training’ analysis run of the sequence processing scripts. The ‘unicell’ category was defined by taxonomy and included all basal eukaryotic lineages that do not include metazoans or plants.

### Proportional Data Analysis

Once sequences for each scat were classified by taxonomy, the proportion of sequences belonging to each taxon in each population was calculated. Population average proportions were calculated from the mean of proportions in individual scats. Comparisons between populations were made by creating two Matrices of pairwise differences among the proportions for each food group within each population. Tests for correlation of the two matrices and their significance were made with a Mantel test [37] implemented with the R package ‘ade4’ [38]. This produced an estimated correlation, r, with a range from complete negative correlation at −1 to complete correlation at 1. An estimate of a p value for this r based on the distribution of r was calculated from 9999 simulated matrices. Comparisons were only made among proportions of food items that were found in both populations at 0.1% or more, which represented multiple sequence counts within each population, but excluded comparisons were no sequence was present in one of the two populations. This procedure tested for changes in ratios of prey items between two populations.

### Sex Identification

A qPCR melt curve assay for identifying penguin sex from scat samples was created by modifying existing assays targeting the Chromodomain helicase DNA binding 1 (CHD1) gene that has size polymorphisms between the Z and W sex chromosomes of most birds. This region has been used in similar assays for many years [39], [40] and our assay targeted a very small region of this, which made it especially successful in amplifying the fragmented CHD1 in penguin scat DNA. A full description of the validation and range of species that this works on has been submitted for publication separately.

Reactions of 10 uL took place on a Roche LC480 thermal cycler. Reactions contained 1 x Light Cycler Probes Master mix (Roche), 1 x EvaGreen, 1 x Bovine Serum Albumin (NEB) and 1 uM of each primer (see Table 2). Amplification conditions were 95°C, 5 min; then 40 cycles of 95°C, 10 s 60°C, 30 s with signal acquisition and 72°C, 10 s. Melt curve conditions were 55°C to 95°C at a ramp rate of 2.2°C per s with 5 signal acquisitions per degree.

### Ethics Statement

Penguin scat samples were collected in the Australian Antarctic Territory with permission of the Australian Commonwealth Government under Australian Antarctic Science permits 2926, 4014, 2722 and 4088. No penguins were handled or approached closely as part of this work as the study material was scat samples collected outside breeding colonies. The collection procedure was approved by the Antarctic Animal Ethics Committee under project permits 2926, 4014, 2722 and 4088.

### Data Accessibility

All data generated in this study, including the raw reads from the IonTorrent sequencer; the scripts used to process them, including example files; and spreadsheets containing the data are available from the Dryad database (http://datadryad.org/) with the doi: 10.5061/dryad.1rf7d.

## Results

### Overall Analysis of Adélie Penguin Diet in East Antarctica

A total of 534 scats were collected in four different years from four locations in East Antarctica as shown in Figure 1. The numbers of samples taken at each site and year are given in Table 1. After DNA was purified from these scats, PCR was performed with a ‘universal’ primer set which amplifies a taxonomically informative amplicon from the 3′ end of the small subunit ribosomal rDNA (SSU). A blocking oligonucleotide was included in PCR to limit amplification of penguin DNA and resultant amplicons. 389 samples (73% of those collected) produced amplicons that were sequenced with the IonTorrent sequencer.

A total of 1.27×10^6^ sequences passed quality checks on the Ion Torrent software and then Q score filtering and forward and reverse primer tag recognition in our scripts. These were derived from 16 runs of 314 chips and 6.5×10^6^ raw reads. Based on classification of sequences to broad taxonomic groups with our purpose written software, we recovered 650,520 sequences representing food items (∼51%); 136,056 sequences from parasites; 164,913 sequences from unicellular eukaryotes and 189,462 sequences that were classified as ‘contaminants,’ which had a sequence database match and includes penguin sequences. 133,943 sequences were unassignable, meaning that they did not have a match in the SSU database. These sequences include chimaeric PCR products, amplicons from organisms without SSU reference sequences and sequencing reads that although they had good overall Q scores they had low quality regions within them. Our assignment of sequences to individual samples involved recognition of tags on both the forward and reverse primers. This strategy of primer tagging to recover amplicons belonging to individual scats is highly effective in other situations where sequencing is accurate in the range that includes the reverse primer tag [20], [21], [31], [32]. However, the 200 bp IonTorrent sequencing kit produced sequences with a high error rate at their 3′ end, which caused large numbers of reads to not be assignable to the individual scat SSU amplicon pools that they were derived from. This strategy was still valuable for allowing multiple samples to be included in each run, but was not especially compatible with IonTorrent sequencing technology at that stage of its development.

**Figure 2:**
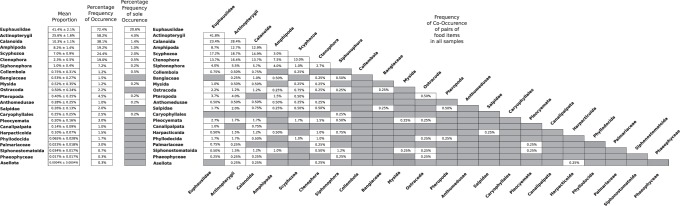
Summary of all diet items. Numerical summaries of DNA based diet analyses for all samples in this study. The food species groups are ranked in order of mean proportion across all samples. The percentage frequency of occurrence of food groups in all samples and the percentage frequency of samples that only had one food item are given. The frequency with which samples in the population had co-occurrence of food items on the Y and X axes of the grid is given. Grey squares indicate no value.

The proportions of food sequences for all scats in this study that belong to each broad prey group are shown in Figure 2. Also shown is the overall frequency of occurrence (FOC) of each food group and co-occurrence of pairs of food groups. These samples were collected at different phases of the breeding season at all locations shown in Figure 1 and Table 1 so grouping them is intended to increase knowledge of the overall food diversity present in Adélie penguin diet. It is important to emphasise that the DNA proportions shown here are not intended to be a direct representation of biomass, energy consumed, or number of individual prey items consumed. There will be some correlations with quantity of sequences and these diet parameters, but biases are certain to exist as with any diet analysis method.

Krill (Euphausiidae) were the most important item in Adélie penguin diet in East Antarctica, making up about 40% of the proportion of the averaged DNA proportions and occurring in about 70% of samples and being the only diet item in 20% of all samples. Bony fish (Actinopterygii) and calanoid copepods are the next most significant diet items and the importance of these three groups correlates with other studies [16], [27]. Interestingly, Calanoida occur with fish (28%) more often than they do with Euphausiidae (23%) despite Euphausiidae occurring more often than fish in the overall diet results. This indicates that some of the detected copepods likely represent secondary predation (i.e. from consumption of fish that have eaten copepods). Amphipod shrimps (Amphipoda), have been identified in stomach contents of Adélie penguins previously [16], but have not been detected at the high relative abundance that we found them in at Mawson in 2010 and 2011. These also occur more frequently along with fish (12.7%) than with krill (8.7%) indicating that some of the penguin amphipod consumption may also be secondary.

The next most important groups are ‘true’ jellyfish (Scyphozoa) and comb jellies (Ctenophora), which have not previously been thought to be common in Adélie penguin diet, although gelatinous species have been recognised as a significant part of the diet of other seabirds [41], [42]. Other groups that have not to our knowledge previously been identified in Adélie diet and are unequivocally diet items include Siphonophora, Anthomedusae, Canalipalpata, Harpacticoida, Siphonostomatoida, Mysida, Asellota and Pleocyemata. The frequency of co-occurrence of prey groups in all scats in this study in Figure 2 shows that some of these rare items occur both with predatory food items such as fish or jellyfish and also with non-predatory groups such as pteropods or copepods. For example, ostracods co-occur with Pteropoda, Pleocyemata, Harpacticoida and Siphonostomatoida, none of which are predatory on ostracods, as well as fish. This indicates that they are likely to be mostly consumed by penguins directly.

Some items are not certain to be food. We took a conservative approach of including items like Springtails (Collembola) and terrestrial plants (Caryophyllales) in the ‘food’ category of the analysis as it is possible that these are consumed, although equally likely that they contaminate the scats before collection. Collembola are common in Antarctic soils and Caryophyllales are the only flowering plants in this region of the Antarctic, so their pollen or other parts may contaminate the scats. These are only minor diet items, so their inclusion doesn’t greatly alter the overall analysis.

No squid DNA was identified in this study. Squid beaks were identified previously in the stomach contents of 19 of 105 dead penguin chicks from Adélie Land [8]. We were surprised by this and wondered whether it was an artefact of the PCR method or the subsequent data processing. To test this, we spiked a sample with squid DNA and added extra squid sequences from GenBank to the local SSU BLAST database. When we ran the samples again, we correctly identified the spiked-in squid DNA. We have also identified squid in albatross diet using the same methodology, so we have some confidence that their absence here represents reality and squid were unimportant in the diet of these penguin populations.

**Figure 3:**
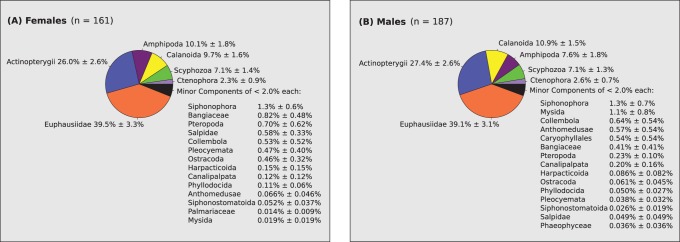
Female and male Adélie penguin diet. A comparison of male and female diet for all samples in this study that produced recognisable food DNA sequences and which had a successful molecular sexing assay result. The pie charts show the estimated mean and standard error of the proportions of DNA sequences from each food group for females (A) and males (B).

The scat DNAs that produced SSU food amplicon results were used as template for PCR amplification of penguin CHD1alleles, which resulted in sex identification of 348 (89%) of the samples. Analysis of all dietary results that also had a sex assigned to them allowed an overall comparison of diet between male and female penguins as shown in Figure 3. This shows a remarkably similar overall diet among male and female Adélie penguins (Mantel r = 0.995, p = 0.0001).

### Comparisons of Diet among Adélie Penguin Populations

**Figure 4:**
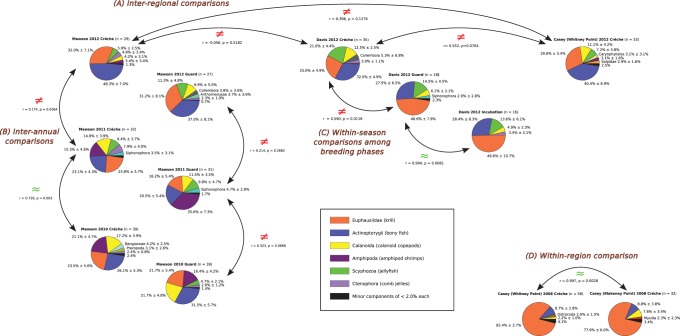
Spatial and temporal comparisons of Adélie penguin population diet. Summary pie charts showing the population means of proportions of diet items for all twelve sample sets analysed in this study. The year and sampling location are indicated above each pie chart with the number of scats analysed in brackets after that. Mean percentages of DNA sequences for each group of food species and the standard error of the estimate are given. Four types of comparison among the samples are made. Comparison (A) is of Inter-regional diet differences among samples from Mawson, Davis and Casey collected during the Créche phase of the breeding cycle in 2012. A smaller-scale spatial comparison is shown in (D) within the Casey region between two samples collected in 2008. Inter-annual comparisons are shown in (B) for samples collected at Mawson among ‘Créche’ samples in 2010,2011 and 2012 and ‘Guard’ samples in 2010, 2011 and 2012. Within-season comparisons are shown in (C) for samples collected at ‘Incubation’,’Guard’ and ‘Créche’ phases of the cycle at Davis in 2012. Results of Mantel tests indicating overall similarity or differences between population diet ratios are indicated.

Changes in relative proportions of DNA sequences were used to infer differences in prey consumption among different sets of samples. Overall differences in prey consumption were inferred by comparing matrices of pairwise ratios among all prey species present in each population. Significant differences as recognised by a Mantel test indicated an overall change in relative proportions of prey items. A summary of all the comparisons made in this way is given in Figure 4. DNA sequence proportions for individual scats can be seen in the population summaries included in the Dryad database entry for this study.

Two spatial scales of diet comparison were made. Comparisons were made among three widely separated sites in East Antarctica at ranges of 700–2100 km separation as shown in Figure 1. In 2012, collections were made at Mawson, Casey and Davis during the Crèche phase of the breeding cycle. The population diets estimated from these locations were substantially different as shown in Figure 4A. Notable differences include a higher proportion of fish (Actinopterygii) consumed at Mawson than at Casey or Davis; and a higher proportion of jellyfish (Scyphozoa) consumed at Davis than at Casey or Mawson. In contrast, population diets were estimated to be very similar from samples collected at two colonies in the Casey region at Whitney Point and Blakeney Point that are only 3.5 km apart as shown in Figure 4D.

Temporal comparisons of population diet were made at two scales. Interannual comparisons were made for samples collected from Mawson at the same phase of the breeding cycle in three consecutive years. This site produced the majority of the amphipod sequences found in this study. Small numbers were also found at Casey but Figure 4B shows that during 2010 and 2011 these were a major diet item during both Guard and Crèche breeding cycle phases at Mawson. However, in 2012 they are almost absent, only being found as a minor component of food DNA in one scat collected during Guard phase.

Comparisons among different phases of the breeding cycle were made at Davis as shown in Figure 4C. The diet during the Incubation and Guard phases of the breeding cycle was similar but changed with the transition to Crèche phase. This apparently represents a decrease in the amount of krill (Euphausiidae) in the diet and an increase in jellyfish (Scyphozoa) and calanoid copepods (Copepoda).

### Detection of Parasites and Eukaryotic Microbes

**Figure 5:**
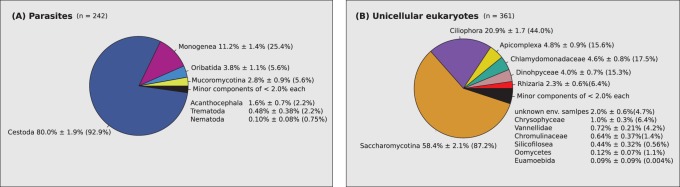
Parasites and unicellular eukaryotes in Adélie penguin scats. Sequences classified as parasites (A) and unicellular eukaryotes (B) identified in all penguin scats analysed in this study. The pie charts show the estimated mean and standard error of the proportions of each sequence type with the percentage FOC in brackets.

The SSU 3′ primer set amplifies an SSU amplicon from a wide phylogenetic range of eukaryotes. Many of these are not food items, but some of the groups are of interest for other reasons. Figure 5 shows metazoan parasites and unicellular eukaryotes identified in all scats analysed in this study. Tapeworm (Cestoda) DNA is present in almost 93% of scats that contained amplifiable DNA and forms a large proportion of the parasite DNA. Monogenea are ectoparasitic flatworms generally known as fish parasites and these may have been ingested with fish, or they may be penguin parasites that are consumed in grooming. Mites (Oribatida) are likely to be penguin mites that either are consumed in grooming or contaminate scats from nests or the body of penguins. The unicellular eukaryote diversity is dominated by Saccharomycotina, which are apparently a large part of the gut microflora.

## Discussion

Adélie penguins and other high trophic-level predators are used for ecosystem monitoring because key population parameters such as breeding success or population size can be estimated conveniently and with reasonable precision [1], [9], [15]. Changes in these parameters are strongly linked to changes in food availability and are thought to reflect changes in ecosystem conditions at lower trophic levels [3]. Breeding success of penguins has been shown to be the most important proxy indicator of changes in availability of krill and fish [28], [43]–[45]. Adélie penguin population size also responds to food availability over longer time frames [29], [45], [46]. The most likely factors forcing changes in Adélie penguin diet are competition with the expanding Southern Ocean fisheries for fish or krill [47], [48]; and changes in prey species composition in response to the effects of current ocean acidification [49] and warming trends [2] that will effect sea ice distribution. Dietary analysis methods that can identify specific changes in population diet are therefore important for understanding the direct causes of changes in Adélie penguin population breeding success or size.

The prevalent methods for studying the diet of penguins are stomach flushing followed by identification of food species present in stomach contents from morphology [50]; and stable isotope ratio analysis [6], [51]. Stomach flushing penguins presents several problems. One is that it is labour intensive so that a limited number of samples can be collected within a season and it is therefore not well suited to fine-scale diet analysis. It also has a clear impact on the animals being studied as handling of the adult penguins can physically harm them and it denies their chicks a meal, which is likely to be detrimental for their survival [52]. The negative impacts of this method have made animal ethics approval for this approach increasingly difficult to obtain. Stable isotope analysis can be performed less invasively but it provides only very broad indications of dietary change that are difficult to interpret because no taxonomic information is produced. It can be used to infer differences between fish and krill dietary dominance because these groups have different trophic levels, but only at very broad temporal or spatial scales and without providing any detail of other prey components [6].

In this study, we identified 23 prey groups of Adélie penguins. Several of these prey groups have not previously been identified in Adélie penguin diet and some of these were significant components of overall population diet. Jellyfish (Scyphozoa), comb jellies (Ctenophora) and bluebottles (Siphonophora) are all soft-bodied and unlikely to be visually identifiable in stomach contents after ingestion. It is possible that identification of their DNA in Adélie scats is the result of secondary ingestion, but some individual scats only contained jellyfish or ctenophores as food items and co-ingestion analysis revealed that co-ingestion was often with animals smaller than jellyfish such as copepods or krill, so we conclude that jellyfish were a primary food item. This is not unlikely given widespread predation on jellyfish by other seabirds [41], [42]. Jellyfish were present as a diet item in all samples sets and ctenophores in all but one (Mawson 2011 ‘Guard’). The importance for ecosystem monitoring of recognising Adélie penguin predation on these gelatinous groups is that they provide a food source that the penguins may switch to when krill and fish are scarce. This may be especially significant in coming years for ecosystem monitoring with Adélie penguins as overfishing of fish in many ocean regions has been demonstrated to cause strong increases in jellyfish abundance [53].

Copepods were a major food item. Co-ingestion analysis indicated that copepods were present in association with fish in the diet more often than with krill, despite krill being consumed more commonly than fish. Krill do not commonly feed on copepods [54], whereas many Southern Ocean fish do and are generally more carnivorous(Clarke *et al.* 1998) [55], [56]. This indicates that at least part of the copepod consumption by Adélie penguins may be the result of secondary predation, but as some Southern Ocean copepods reach 12 mm in length, primary predation is still very likely.

Identification of penguin sex associated with each scat demonstrated a remarkable overall similarity in diet between the sexes, although this was clearly for an average of all samples. Sex-specific feeding has been demonstrated in other studies of Adélie penguins [7]. It would be interesting to see if the strong similarity we found for all samples in this study is also found at a local population scale and collection of larger sample sizes within a season and breeding phase could allow this to be addressed. However, our overall result does indicate that for the purpose of ecosystem monitoring, it is reasonable to group male and female scat samples for diet analysis.

Spatial variation in diet was evident from synchronous sampling of widely separated populations. In the Crèche breeding phase of 2012, there were clear differences among the three sites sampled in the proportions and frequency of consumption of important dietary items such as fish, copepods and jellyfish, although consumption of krill was fairly constant. Sampling of two colonies on adjacent headlands at Casey in 2008 produced very similar dietary results, which is to be expected as the colony separation is far less than the foraging trip range of the penguins and prey availability is unlikely to be significantly different over such small distances. Similar observations have been made for Adélie penguins in the Ross Sea where foraging ranges among colonies overlap [2], [16]. This suggests that multiple sample sets are not required to characterise diet in one region for ecosystem monitoring purposes.

Temporal variation in diet was observed at two sites. At Mawson, inter-annual diet variation was most clearly identified where Amphipoda were a major diet component in both Guard and Crèche breeding cycle phases in 2010 and 2011, but were almost absent in 2012. This is a clear example of the ability of Adélie penguins to switch to different prey groups. Within-season variation in feeding was indicated at Davis, where a fall in krill abundance relative to fish, jellyfish and copepods was detected between the Guard and Crèche breeding phases, whereas Incubation and Guard phase were very similar. Foraging trip length is known to increase in the transition from Guard to Crèche phase, where chicks form groups that are guarded communally, which increases time available to parents for foraging [15], [16]. Changes in foraging behaviour may explain the relative increase of fish and jellyfish consumption indicated in the Crèche samples.

A common goal of diet analyses is to quantify the different diet components by biomass, energy content or number of individuals consumed. However, all diet analysis methods have inherent biases and DNA based diet analysis is no exception. One of the known biases is differences among food species in PCR product strength per unit of biomass consumed [19]. DNA-based methods will never provide absolute quantification of individual prey species in the same way that stomach content analysis can (although still with biases), where total stomach contents can be weighed [57]. There is, however, potential for relative quantification with DNA based analyses and some authors have reported very consistent PCR product intensities among closely related groups of food items [21]. Even if there are biases in PCR product: ‘real’ food consumption ratios among food items, the type of proportional population diet estimates presented here can detect change in overall consumption of food items between populations. This procedure allows change to be detected between sample sets even if some food items are consistently over or under represented by proportion. PCR and next-generation sequencing has a large quantitative response range, so frequency of occurrence analysis is likely to be misleading as tiny or large amounts of a given food species amplicon will be detected and scored in the same way. This bias is exacerbated in situations where there is a large size range among prey as in this case, where a copepod may weigh 10,000 times less than a fish or a jellyfish. Diet analysis and interpretation with any method is complicated, so a range of different approaches are used in different situations. The approach we chose is aimed at identifying major changes between sample sets in consumption of prey groups, which is appropriate for population diet monitoring.

When applied to ecosystem monitoring, relative DNA quantification can identify major changes in relative prey abundance and will provide taxonomic identification for the taxa that change. The DNA fragment that we used here gives family or higher level taxonomic identification, but species-level DNA identification in penguin scats has already been demonstrated for krill [5] and bony fish [19], the two most important prey groups identified here. Adding these molecular assays would be especially useful for investigating the proportions of the three major krill species available to Adélie penguins, *Euphausia superba, Euphausia crystallorophias* and *Thysanoessa macrura*. It would also be informative to investigate the proportion of fish predation that is on myctophidae, *Pleuragramma antarcticum* or other fish. Fish are the prey group that is most likely to be affected by the predator blocking oligo in the SSU PCR because their SSU sequence is closest to that of penguins. Even though none of the Southern Ocean fish are thought to have a complete match to the blocking oligo, even partial matches may bias the results. This is another reason to assess fish consumption with a secondary primer set.

A major advantage of being able to use scats as a substrate for diet analysis is the ease with which large sample numbers can be collected in short time periods. Despite only 73% of collected samples returning dietary information from this molecular assay, we were still able to generate detailed diet information for short time periods with only a small amount of field time required. It would certainly be possible to analyse diet over much shorter time periods of weeks, or even days, at large colonies where fresh penguin scats are abundant. The ability to analyse large numbers of samples with this method revealed a significant local temporal and spatial abundance of Amphipoda, which would have been missed in a more restricted sampling regime. Fine-scale temporal/spatial diet studies could be especially useful for investigating fishery-driven diet changes as sampling regimes that target the right locations and times could identify dietary shifts associated with known fishery activity.

The SSU PCR and subsequent bioinformatics pipeline described here automatically returns information of parasites and eukaryotic gut microbiota. While this was not the primary goal of this study, it could be used for estimating parasite prevalence as a parameter for monitoring population health. A more thorough study of the gut microbiota that included bacterial and archaeal characterisation would be especially interesting when considered in concert with the dietary information. DNA based diet analysis with this method should allow many unanswered questions to be investigated such as comparisons of chick and parent diet; comparing diet of breeding and non-breeding penguins; and collecting scats from the sea ice zone in winter to further understand the diet of Adélie penguins throughout the year. Our diet analysis approach is directly applicable to diet analysis for most birds and mammals without modification because the blocking primer works for most tetrapods and the SSU database and scripts for processing the sequence data will identify almost all likely food groups. This makes the approach especially valuable for dietary comparisons among different species. The SSU primers amplify from animals, plants and fungi so the method will provide dietary information for animals with diverse diets.

### Conclusions

Ecosystem monitoring utilising DNA based diet analysis of top level predator diet is a new approach that has not been widely tested. Our results indicate that a broad-scale DNA based analysis can identify changes in Adélie penguin diet at regional, inter-annual and inter-season scales. The diet of this species is far more diverse than has previously been revealed by gut content and stable isotope analysis. It is important to be able to detect all significant components of Adélie penguin diet as fisheries for krill and bony fish are likely to cause a shift to some of these groups such as jellyfish and comb jellies. Scat collection is a simple and efficient field collection procedure that allows a wide range of questions on diet to be addressed. Analysis of food DNA with the broad-scale PCR and sequencing approach presented here is efficient once established in a laboratory and can be supplemented with other tests for penguin gender, or for species-level food identification. We anticipate that the sort of approach we present here could make a significant contribution to ecosystem monitoring programs and we show how it might be useful for CEMP here. The methodology used here is directly applicable to monitoring the diet of any bird or mammal and could be used to address a variety of ecological questions.
